# Urine disinfection and *in situ* pathogen killing using a Microbial Fuel Cell cascade system

**DOI:** 10.1371/journal.pone.0176475

**Published:** 2017-05-02

**Authors:** Ioannis Ieropoulos, Grzegorz Pasternak, John Greenman

**Affiliations:** 1 Bristol BioEnergy Centre, Bristol Robotics Laboratory, University of the West of England, Bristol, United Kingdom; 2 Centre for Integrative Biology CIBIO, University of Trento, Povo, Trentino, Italy; University of Notre Dame, UNITED STATES

## Abstract

Microbial Fuel Cells (MFCs) are emerging as an effective means of treating different types of waste including urine and wastewater. However, the fate of pathogens in an MFC-based system remains unknown, and in this study we investigated the effect of introducing the enteric pathogen *Salmonella enterica* serovar *enteritidis* in an MFC cascade system. The MFCs continuously fed with urine showed high disinfecting potential. As part of two independent trials, during which the bioluminescent *S*. *enteritidis* strain was introduced into the MFC cascade, the number of viable counts and the level of bioluminescence were reduced by up to 4.43±0.04 and 4.21±0.01 log-fold, respectively. The killing efficacy observed for the MFCs operating under closed-circuit conditions, were higher by 1.69 and 1.72 log-fold reduction than for the open circuit MFCs, in both independent trials. The results indicated that the bactericidal properties of a well performing anode were dependent on power performance and the oxidation-reduction potential recorded for the MFCs. This is the first time that the fate of pathogenic bacteria has been investigated in continuously operating MFC systems.

## Introduction

A Microbial Fuel Cell (MFC) is a bioelectrochemical reactor in which organic compounds in the feedstock are oxidised in the anodic chamber to produce carbon dioxide, protons and electrons (in the form of NADH/NADPH) within the microbial cells. Feedstock fuel (utilisable substrate) can include a wide range of compounds, from acetate and other low molecular weight monomers, including sugars right up to particulate macromolecules and complex real world mixtures such as sludge and urine, under anaerobic conditions in the anode chamber. Utilisation of complex macromolecules which could be present in urine [1] implies the abundance of hydrolytic activity around and within the anodic biofilm. Electrons derived from the NADH redox reactions within the cell are transported to the anodic electrode (via direct conductance or via chemical redox mediators) and then travel through the external circuit to the cathode within the cathodic chamber, while protons generated from the biofilm on the anode pass through a membrane, which separates both chambers [2]. The use of this technology has attracted increasing interest in recent years, and several scaled-up applications have been successfully demonstrated, for treating waste products (as microbial electrolysis cells or MEC) [3], [4] as well as for cleaning waste and producing electricity (as true Microbial Fuel Cells) [5]. The cost-effectiveness of the MFC technology [6], [7] has also been demonstrated.

Although the MFC technology has seen significant scientific development over the last three decades, implementing this technology in real-world applications requires extensive studies on health and sanitation hazards. These concerns have rarely been addressed.

An important part of the health risk assessment for wastewater treatment technologies consists of determining the fate of enteric pathogens *in-situ* and *ex-situ* of a treatment process. Insufficient sanitation, together with unavailability of improved water systems, leads to hundreds of thousands of deaths each year, particularly in Sub-Saharan Africa [8]. Therefore, the MFC technology, which could be used in remote, off-grid areas of Developing World countries, to treat wastewater and generate electricity [9], [10], [11], offers great promise. Although pathogens are likely to be more commonplace in fecal sludge it is common practice to separate the solid fraction from the top liquid fractions in sludge, and the liquid fraction is usually dominated by urine. Moreover, the liquid fraction can spread pathogens greater distances from the source of contamination.

There have been some reports already published regarding the disinfection potential of MFC-driven processes. Nevertheless, these studies were focusing on the disinfecting properties of the synthesised catholyte, which could be a result of oxygen reduction to H_2_O_2_ [12] or electro-osmotic drag occurring in the MFCs. The H_2_O_2_ synthesis occurs spontaneously at the cathode and was successfully employed to disinfect the effluent of a wetland system. Treating the effluents with H_2_O_2_ solution resulted in a significant decrease of total coliforms [13]. Another approach for disinfection with the use of catholyte was described by Jadhav *et al* [14]. The authors supplied the cathodic chamber with sodium hypochlorite for the simultaneous improvement of power output and disinfection, which was performed by recirculating the anolyte through the cathodic chamber. A more recent study showed that highly alkaline catholyte produced in ceramic MFCs, also possesses strong disinfecting properties; its application resulted in significant decrease of metabolic activity of *Escherichia coli* [15] chosen as a representative enteric pathogen. The production rate and properties of the catholyte are dependent on the properties of the ceramic separator, as well as the power output. It has been reported that the pH of the catholyte and its disinfection strength is positively correlated with the ceramic membrane thickness [16]. Moreover, pH alone can be favourable to growth, when at or close to neutral (pH 6.0–7.5) whilst much higher (>pH 8.0) or much lower levels (<pH 5.5) may contribute strongly to bacterial survival or killing.

The catholyte is one example of the numerous applications that makes MFCs a platform technology, and one that offers great promise for further investigation. However, the exposure of MFC anodes—or any bioelectrochemical system—to pathogenic organisms in a real environment, is a parameter that has not been assessed before, and the hypothesis is that exogenous microorganisms entering an anodic MFC chamber, will be out-competed by the established electroactive community, if the latter is thriving under, or close to maximum power transfer conditions.

The aim of this study was to investigate the fate of one of the most important members of the *Enterobacteriaceae* family, namely *Salmonella enterica* serotype *enteritidis*. This rod-shaped gram-negative species, which may originate from sewage contamination [17], may cause food-borne diseases. This species was introduced into an MFC cascade system treating human urine, to determine the anodic killing efficacy when operating in continuous flow conditions. This study used bioluminescent reporter strains to measure the rate of killing in situ and used viable counts on selective recovery media to demonstrate that urine can be efficiently disinfected by the MFC cascade system.

## Materials and methods

### MFC construction and operation

Ceramic earthenware cylinders were used to build open to air cathode, small scale MFCs. The ceramic (Scientific & Chemical Supplies Ltd, UK) material was used both as the proton exchange membrane and body of the MFC. Each ceramic cylinder was cut to maintain the internal volume of empty MFCs equal to 11.4 mL. Carbon fiber veil with a carbon loading of 20 g/m^2^ was used as the anode (PRF Composite Materials, Dorset, UK). Anodes were cut into rectangles of dimensions 9×28 cm and folded to obtain a total surface area of 252 cm^2^ and threaded with a plain Ni-Cr wire (Ø0.45 mm, Scientific Wire Company, UK), which was used for connection. The cathodes were made of conductive graphite paint as described by Winfield *et al*. [18]. In brief, petroleum spirit was used to dissolve the polyurethane rubber coating (PlastiDip, Petersfield, UK) and mixed with graphite (Fisher Chemicals, UK) in a 1:3 (plastidip:graphite) ratio. Two layers of conductive paint (carbon loading of 35.02 mgC/cm^2^) were applied on the surface of the ceramics and supplied with a nickel-chromium mesh used as a current collector (20x20, 0.18 mm). The total carbon loading for the cathode was 0.851 gC, and the projected surface area was 24.18 cm^2^.

The 3D printed Nanocure^®^ RCP30-resin lid designed with inlet and outlet tubes was used as the front of the MFC, whilst a transparent acrylic lid (3 mm thick) was used to cover the other side of the chamber. The RCP30-resin lids were designed using SolidWorks 2013 software and printed with Perfactory 4 3D printer (Envisiontec, Germany). Both lids were assembled with the MFC by a plain nylon screw (Ø3 mm, RS, UK). The detailed schematic representation of individual MFCs has been described previously [19].

During the first trial, additional 2 cm^2^ of carbon veil wrapped around Ni-Cr wire were introduced through the channel in the lid to the anolyte chamber. These removable additional ‘anodes’ had a direct physical and electrical contact with the main anode in the MFCs and allowed the formation of the anodic biofilm. These removable anodes allowed aseptic sampling of biofilms (by *in situ* detachment) to monitor the incorporation and survival of *S*. *enteritidis* cells in the anodic biofilm.

### Experimental setup

**Fig 1:**
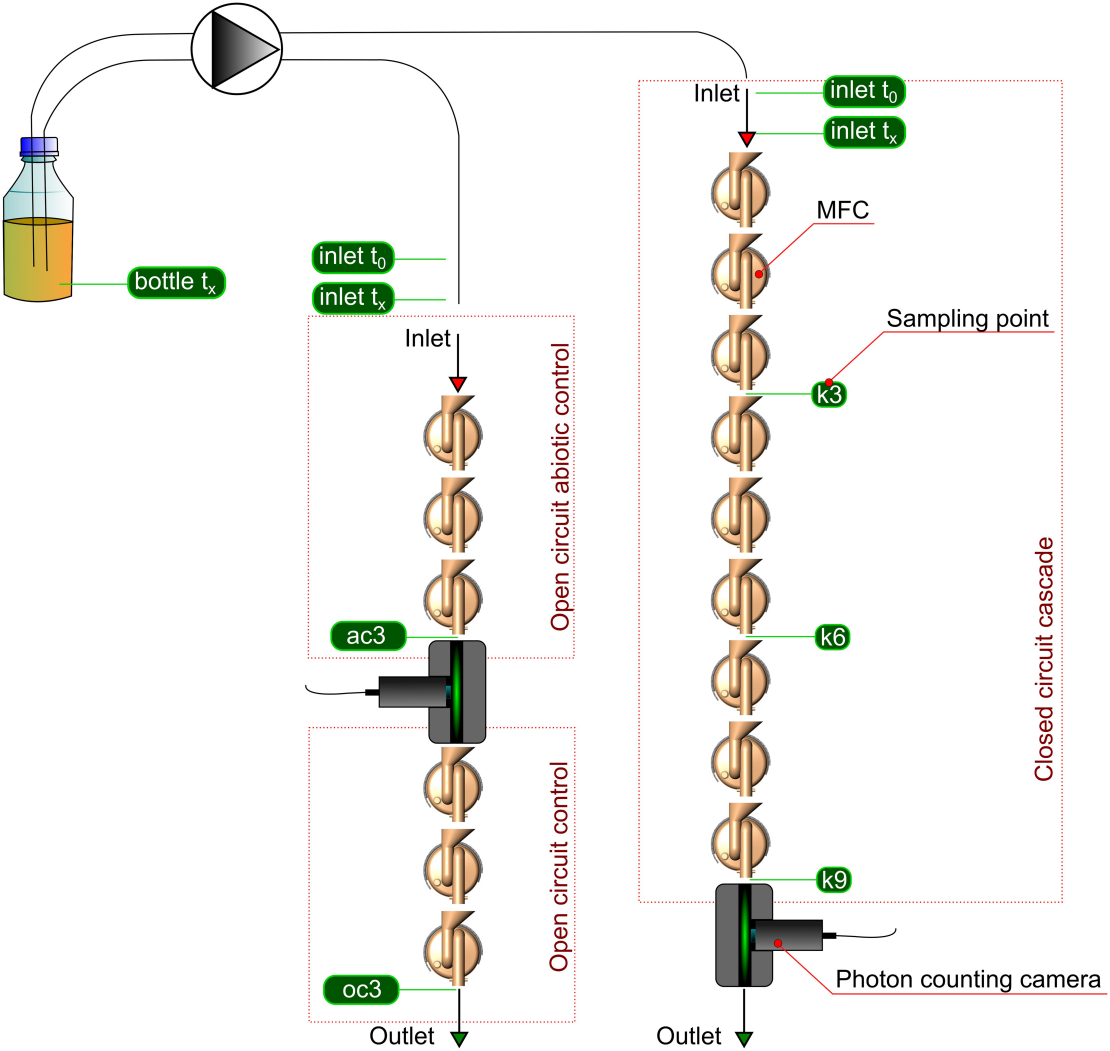
Schematic representation of the experimental setup.

Two individual trials were run over the experimental period. The first trial consisted of a cascade of 9 MFCs operating in closed circuit (CC) conditions and a separate cascade of 3 MFCs operating in open circuit (OC) conditions (Fig 1). Both the CC and OC MFCs were inoculated with the mixed bacterial community derived from anaerobic activated sludge (Saltford Scientific Laboratory, Wessex Water, Bristol UK).

The second trial consisted of the same two cascades as trial 1, with an additional control with 3 OC/abiotic control MFCs. This additional control cascade was setup such that its liquid output was flowing into the input of the OC/biotic control cascade. The abiotic control MFCs were disinfected prior to the experiment by using 70% ethanol solution, followed by washing with sterile water and drying at 60°C for 1 hour. All of the MFCs were separated by physical air gap between the cells to avoid any conductive bridging between the anodes.

To estimate the killing potential against *S*. *enteritidis* and monitor its metabolic activity in real time, a flow cell supplied with H10720 photosensor module (Hamamatsu Photonics K.K., Japan) was used. The sensor was introduced after the 9^th^ MFC in CC cascade (trial 1) and after the 3^rd^ MFC in the OC abiotic cascade (trial 2).

Fresh neat human urine (collected not later than 24 hours before the trial and stored at 4°C) was used as a fuel and supplied to the cascades by using a multichannel peristaltic pump (Watson Marlow, USA) at a constant flow rate of 0.90 L/d.

The external load connected to each MFC, was 1000 Ω for the initial 11 days of operation and 250 Ω afterwards for the rest of the experiment. The cascade was fed with fresh human urine as the fuel. Before the trials, the CC and OC MFCs were operated for 167 days in order to fully allow the maturing of the anodic biofilm and to demonstrate the feasibility of disinfection in a well-established MFC system.

### Introduction of *Salmonella enteritidis* strain

The *S*. *enteritidis* strain was obtained from the collection of the University of the West of England, the serotype designation was validated by serotyping [20]. The strain was carrying the pBBR1MCS-2 plasmid derivative containing the luxCDABE operon of *Photorhabdus luminescens*. Prior to the experiment, the strain was subcultured in LB media containing kanamycin (10 μg ml^-1^) as the selective agent and incubated overnight in 37°C. Subsequently, when the optical density at a wavelength of 600nm (OD_600_) reached 1.0, 15 mL of the culture was centrifuged, washed twice with a 0.9% NaCl solution, re-suspended in 50% glycerol solution, and stored in -20°C until the start of the experiment. To inoculate urine with *S*. *enteritidis*, cryopreserved bacterial cultures were centrifuged and re-suspended in 1 L of neat urine.

### Monitoring the disinfection of urine

To estimate the killing potential of the MFCs in real time, the signal obtained from the photosensor was calibrated with the corresponding signal from the tube luminometer GLOMAX, 20/20 (Promega, USA). The signal was therefore given in Relative Luminescence Units (RLU). Moreover, at the end of experiment, the samples were collected from all of the sampling points and their luminescence was assessed using a standard benchtop luminometer. In addition, at the end of trial 1, small (2 cm^2^) anode pieces were removed from the anolyte chambers and analysed in the same manner after resuspending the biofilm by sterile rod and vortexing (3 min). The quantity of Colony Forming Units (CFU) was assessed using XLD agar (Oxoid, UK). The pH and ORP were measured with Orion Dual Star pH meter (Thermo Fisher Scientific, USA).

The log reduction (LR) of colony forming units, as well as log reduction of bioluminescence intensity was calculated using the following formula:

$$LR=\text{log}\left(\frac{A}{B}\right)$$

Where:

A—number of viable microorganisms or bioluminescence intensity before treatment,

B—number of viable microorganisms or bioluminescence intensity after treatment.

The standard deviation was calculated as described by Zelver *et al*. [21]:

$$SD_{LR}=[(S_{A}^{2}/n_{A})+(S_{B}^{2}/n_{B})]$$

Where:

S_A_ and S_B_—the sample standard deviations of the log reduction values for samples before and after treatment, respectively;

n_A_ and n_B_−number of replicates in population before and after treatment, respectively.

### Data logging and processing

MFC performances, as well as the signal from the photosensor were recorded using a Picolog ADC-24 Data Logger (Pico Technologies, UK), with the data logging sample rate set to 3 minutes. The current was calculated according to Ohm’s law: I = V/R, where V is the measured voltage in Volts (V) and R is the value of the external resistance. The power output P in Watts (W) was calculated using equation: P = I x V. Experimental data were processed using Microsoft Excel 2010 and plotted by GraphPad Prism 5.0 software.

### Statistical analysis

The LR data was analysed using Shapiro-Wilk normality test and t-student test (α = 0.05) to determine the significance of difference between the means. All statistical analysis was performed using R statistical environment.

### Ethics statement

This research involved the use of human urine. The appropriate written consent was given by all individuals participating in the study. This research was approved by NHS (12/YH/0493) and University of the West of England Research Ethics Committee (112207).

## Results and discussion

**Fig 2:**
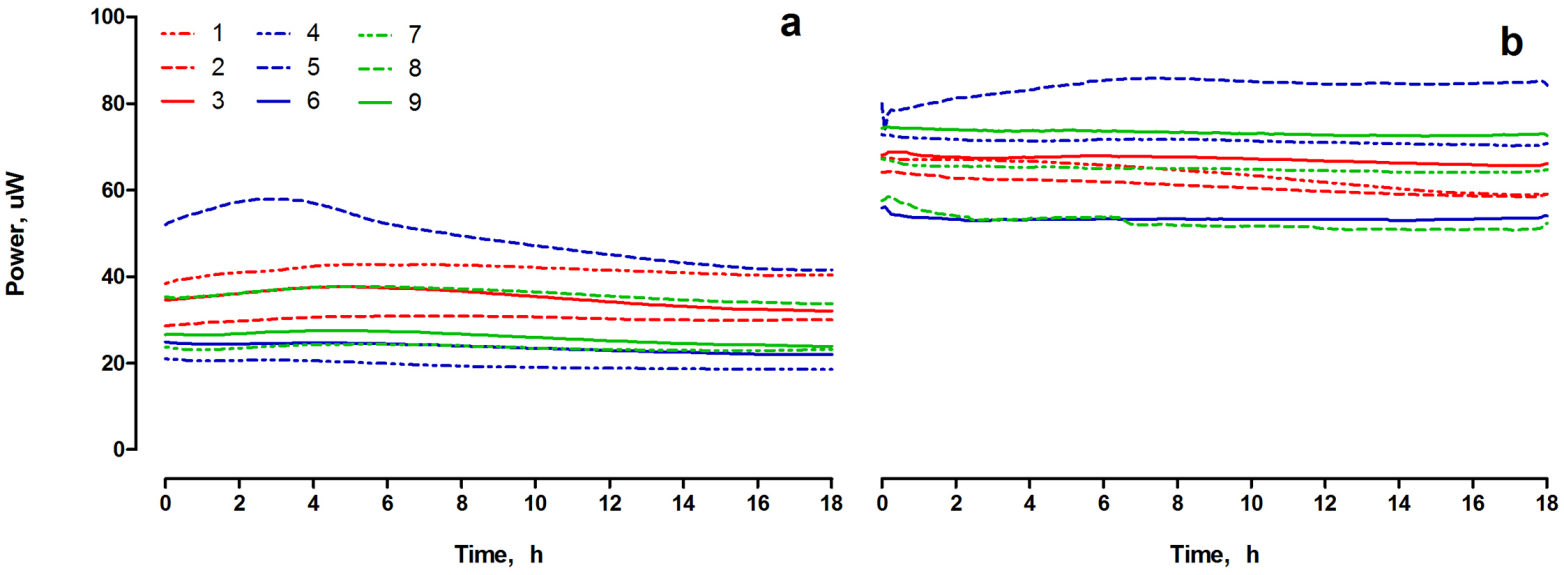
Temporal power performance of the individual MFCs in the cascade system observed during the first (a) and the second (b) trial.

From previous work, it was established that this type of MFCs could reach power output levels of the order of 105.5±32.2 μW [19]. However, during long-term operation, undesirable cathodic biofilm formation caused deterioration of performance. Therefore, the first trial described in the current study, was performed when MFCs produced only 31.2±9.2 μW (Fig 2), which corresponds to 29.5% of the best performance. During the second trial, the biofilm formed at the cathodes was removed, thus the power recorded over the experimental period increased to 65.3±9.3 μW. In both trials, the power performance recorded for individual MFCs was stable and indicated that the fuel supplied to the MFCs, at constant flow rate and in controlled temperature conditions, was utilised at constant reaction rates. The stable power output was the result of the stable metabolic rate and cell population number [22] giving constant electrochemical conditions, which in turn helped to stabilise the whole system, rendering it reliable for investigating the disinfection efficacy of the target reporter species.

**Fig 3:**
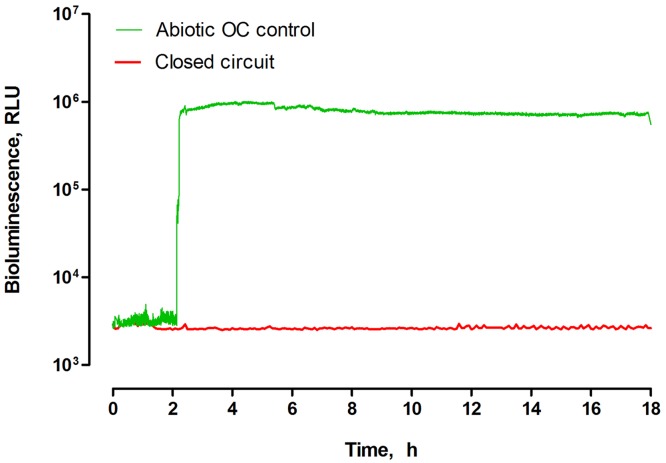
Real-time bioluminescence recorded for the closed-circuit MFCs (after 9^th^ MFC in the cascade) and abiotic open circuit control MFCs (after 3^rd^ MFC in the cascade).

**Fig 4:**
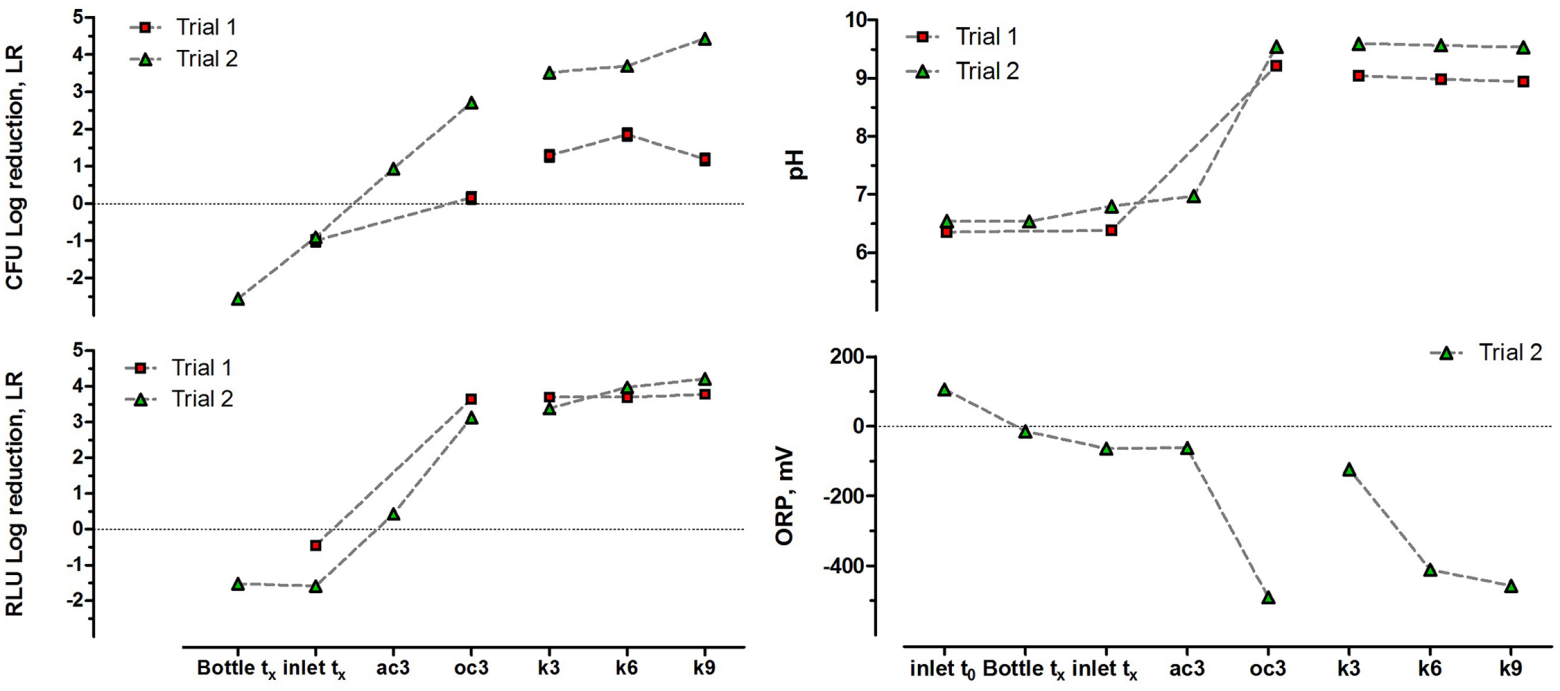
Changes in bioluminescence intensity, bacterial viability and physical-chemical parameters of anolyte. **Log reduction (LR) was calculated using Inlet t**_**0**_ **as the reference point**. LR datasets are represented by an average of 3 replicates ±SD. Closed circuit MFCs were marked by gray circles. The star * symbol indicates the first trial, while labels without symbol indicate the second trial. The data between oc3 and k3 are shown disconnected, since the two cascades were independent (see Fig 1), i.e. the effluent from oc3 did not flow into k3. ORP data are shown for Trial 2 only, due to technical problems of measurement during Trial 1.

The luminescence observed for the whole experimental period did not exceed 3.93×10^3^ RLU. In contrast, a strong signal reaching 1.73×10^6^ RLU was recorded when the photosensor was introduced to the abiotic control (Fig 3). The immediate response of the sensor was observed after the second hour of the experiment, when all of the MFCs in the triplet were fully filled with urine, allowing the treated urine to pass through the sensor chamber. Therefore, the real-time monitoring of bioluminescence intensity with the photosensor introduced in two different sampling points, revealed that the environment within the closed circuit MFCs, was indeed hostile to the pathogens, suppressing microbial activity of exogenous bacteria. The bioluminescence reaching up to 1.73×10^6^ RLU in the case of the abiotic control, indicated that none of the materials used to construct the MFCs, nor the conditions occurring in abiotic OC MFCs were toxic against the *S*. *enteritidis* strain. Although the data from the photosensor were generated from two cascades with a different number of units, the luminescence results were confirmed by analyzing the samples collected from both cascades (3^rd^ unit in each cascade), using the benchtop luminometer (Fig 4).

Monitoring the microbial activity and viability in all of the sampling points, as well as during the inoculation of the MFCs with *S*. *enteritidis* allowed the determination of the log-reduction (LR) values for the above-mentioned parameters. In both trials, a positive effect (negative LR values) on microbial viability (CFU) and metabolic activity (reflected by RLU—bioluminescence being dependent on metabolic rate) was observed as a result of 18 hours of incubation in batch culture (bottle t_x_). Moreover, only negligible difference was observed when comparing RLU LR values of batch culture (bottle t_x_) and the inlet to the cascade (inlet t_x_). The differences recorded in the CFU LR values were probably the result of experimental variance in sampling bacterial cells undergoing sedimentation in batch culture. This demonstrated that there was no disinfection effect prior to entering the cascade system, that might have been caused by mechanical pressure derived from peristaltic pump or redox reactions occurring during the residence within the silicon tubing. Similarly, only negligible negative effects (positive LR values) were observed, when the pathogenic cells were processed through the abiotic OC cascade (ac3). The retention of pathogenic cells in abiotic MFCs may have initiated the adsorption, sedimentation and biofilm formation mechanisms.

It is known, that *Salmonella* species are able to form a biofilm structure on various types of substrata [23], [24], thus some positive effect (decrease of viability) may have been caused by the accumulation of its metabolic by-products, or simply by attachment of the dead or dying bacterial cells to the biofilm. On the other hand, when the biofilm was removed from the 2cm^2^ of anode at the end of trial 1, none of the viable *Salmonella sp*. cells were detected neither in open circuit nor closed circuit MFCs (Table 1). Similarly, the observed LR values for luminescence were indicating that none of the *Salmonella enteritidis* cells were incorporated to the matured biofilm.

**Table 1:** Monitoring of viability and luminescence of *S*. *enteritidis* on the biofilm surface.

RLU LR				CFU LR			
Average	SD	Average	SD	Average	SD	Average	SD
179.3	10.5	5.03	0.02	0	0	>7*	na
228.7	42.6	4.93	0.06	0	0	>7*	na
323.0	58.8	4.78	0.05	0	0	>7*	na
336.7	61.8	4.76	0.06	0	0	>7*	na

*the LR result is shown based on the calculation that 1 CFU would give 7.09 LR. The LR values cannot be calculated when the CFU = 0, na—not applicable.

The log-reduction of RLU value calculated for the abiotic OC cascade (ac3) was 3 orders of magnitude lower than that from the closed circuit MFCs, which is also consistent with the results obtained from the real time luminescence monitoring. Nevertheless, a significant (p<0.05) disinfecting effect was observed for the biotic OC control (oc3). The RLU LR values reached 3.13±0.02 LR and 3.64±0.04 LR in two individual trials, while the CFU LR observed for Trial 2 was equal to 2.71±0.05 LR and 0.16±0.15 LR for Trial 1. In all cases, the LR values were still lower when compared to those observed from the closed circuit MFCs, showing that the killing potential of OC MFCs was much lower than that of the CC MFCs. The difference in CFU LR values when comparing both trials indicates that perhaps the weak disinfecting properties observed from the biotic OC control, could have been the result of lytic or hydrolytic biochemical reactions, which were dependent on the length of time that individual MFCs were running for. Trials 1 and 2 were performed in two different periods of time, resulting in two different power performance levels (as shown on Fig 2). The cathode regeneration procedure [19], was not carried out for the OC MFCs, thus a significant deterioration of the electrodes could have affected the overall OC MFC environment and biofilm metabolism. The ORP values, reaching -490.7 mV (vs SHE), which is the highest negative ORP observed in this study, indicated good dynamic/electrochemical conditions within the OC MFCs (Fig 4). The sub-optimum power output conditions occurring in the CC MFCs (31μW Trial 1 & 65μW Trial 2 vs 105μW max recorded [15]) may have allowed antagonistic (to electron transfer) fermentation processes. Such conditions could have a negative effect on the killing efficacy, suppressing the overall capabilities of the MFCs.

Although the killing effect on *S*. *enteritidis* was observed for the biotic OC control, the CFU LR values observed for the corresponding closed circuit MFCs were significantly higher (p<0.05). Moreover (with one exception), the LR values were increasing after the treatment in each triplet of CC MFCs, reaching up to 4.43±0.04 LR at the end of the cascade. The pH of urine increased from pH 6.8 (when fresh) to as high as pH 8.94–9.59 thus helping to antagonize the growth of *Salmonella* in all of the inoculated, biotic MFCs, while the ORP, monitored for Trial 2 indicated a highly reducing environment. Moreover, a linear decrease of ORP was noticed along the CC cascade. These values of CFU LR are in agreement with those observed when the hydrogen peroxide derived from cathodic reactions was used against coliforms [13].

It is therefore hypothesised that the cascade effect was creating favourable conditions for the removal of pathogenic species from urine by the sequential increase of the reducing force, along with the increase of the pH. The good decrease of the ORP force and increase of the pH, together with the lower LR observed for the OC biotic control (oc3) suggest that ORP and pH were two important factors influencing the killing potential of MFCs. However, although direct effect of ORP and pH on pathogen inactivation was observed, the LR values achieved for closed circuit MFCs were higher than the open circuit controls in all trials. The closed circuit MFCs reached higher LR values in comparison to OC MFCs for which the recorded pH was comparable or even higher (Trial 1 –Fig 4) than for the closed circuit MFCs. Therefore, the results suggest that the increase of pH, caused by the urea hydrolysis was not the only factor contributing to the inactivation of the pathogens. It is assumed that the bioelectrochemical reactions generating electric current were introducing additional stress mechanisms against the pathogenic cells, thus increasing the disinfection effect during the treatment of human waste in MFCs.

**Fig 5:**
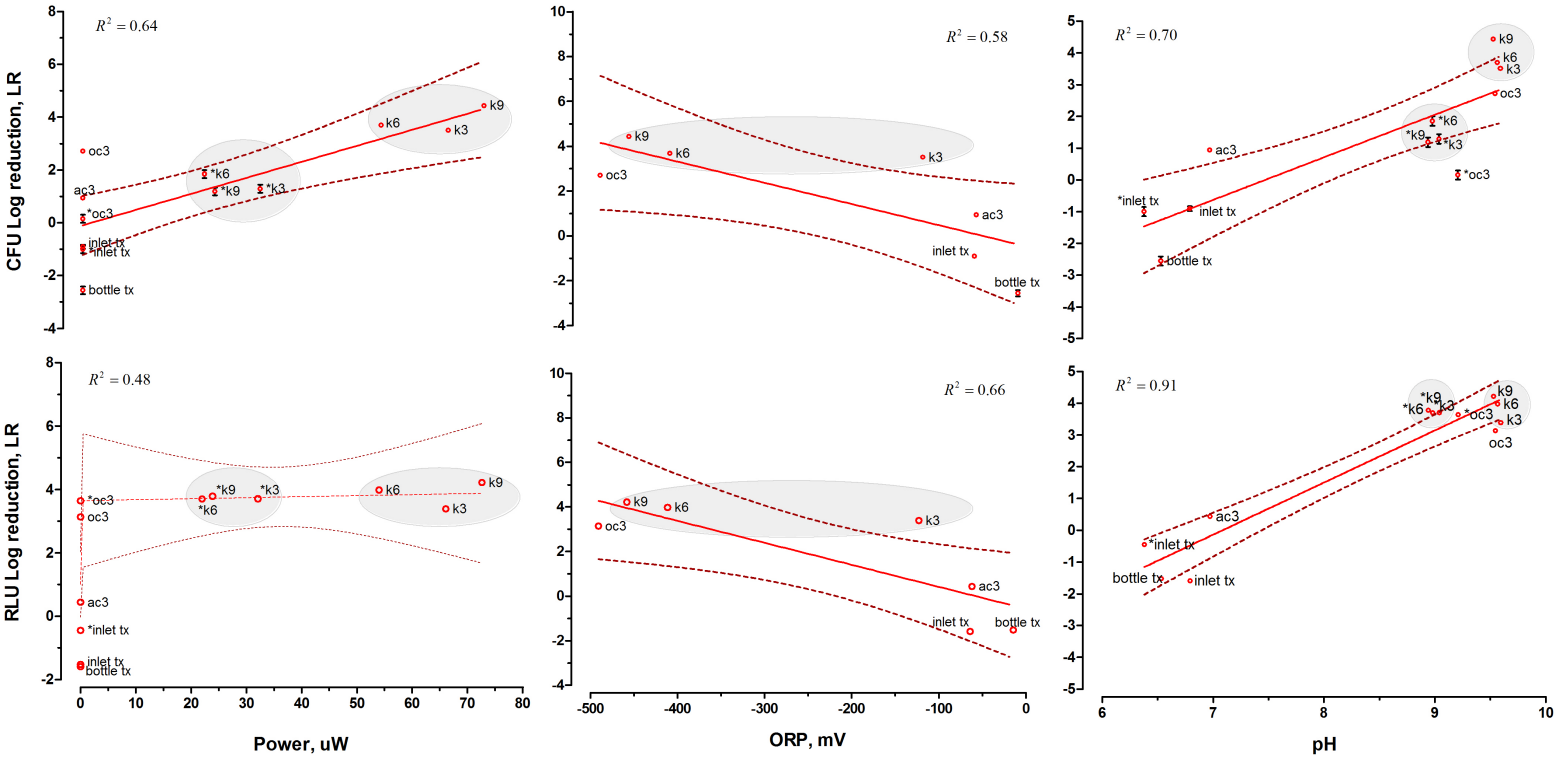
Relationship between power and oxidation-reduction potential with killing efficacy.

To further investigate this hypothesis, linear regression models were tested for ORP, power and LR variables (Fig 5). The highest correlation coefficients were calculated when the effect of power generated by the MFCs on CFU:LR and ORP on RLU:LR were investigated, respectively. These results indicate that although both factors were well describing the LR variables, the change in ORP had a more marked effect on the metabolic activity of the pathogens, whilst power output had higher impact on the viability of pathogenic bacteria. It is therefore concluded that both of these factors played a role in creating a hostile environment for the pathogenic bacteria. The results also indicate that oxidising the urine constituents in current-generating pathways have induced a killing effect when compared to the non-current generating pathways occurring in both types of OC controls.

The negative ORP was driven by the current-producing reactions. The decrease of metabolic activity observed by the decrease of the bioluminescence was followed by a decrease of viability. Such a decrease in viable counts of *E*. *coli* was also observed, when the negative potential was artificially supplied to the carbon fiber electrode, resulting in over 3 log-fold reduction [25]. It is therefore assumed, that the negative anodic potential created in MFCs treating urine may lead to the inhibition of the respiratory chains of bacteria and consequently, death of the cells.

The decrease of the viability of the representative serovar of *Salmonellae* observed in this study was of a higher magnitude, when compared to conventional wastewater treatment plants. In a comprehensive study reported by Koivunen *et al*. [26] the authors have identified 32 different *Salmonella* serovars and recorded removal efficiency of 2 and 3 log units during treatment in biological-chemical reactors and tertiary filtration units. Increased killing efficiency was observed when UV or ozone were used to treat wastewater [27]. The disinfection efficiency observed in this study is similar to the more recently described method of electroporation using a conductive nanosponge [28]. These authors recorded a disinfection efficiency reaching up to 6 log-fold reduction of enteric bacteria (including *Salmonella enterica* serovar *typhimurium*). Nevertheless, the voltage externally applied to the anode was 2 orders of magnitude higher than that produced by the MFCs used in this study and an additional disinfectant was used to induce the killing process. It is possible that the power production in MFCs may have a similar effect on pathogenic bacteria. The electrochemical process described herewith, may lead to the increased uptake of ionic species to the interior of bacterial cells.

It is also assumed that the kill rates observed in this study may have also resulted from the formation of ionic-redox chemical species that led to negative ORP. In addition, the role and contribution of other potential bacteriocidal mechanisms (e.g. lytic enzymes, antibiotics, bacteriocins or other toxic molecules) has not been investigated in the present study. Considering that closed circuit MFCs have shown significantly higher killing efficiency, it is concluded that the production of electric power resulted in changing both the physico-chemical parameters of urine and influenced the integrity of the bacterial cells, leading to a high killing efficacy in a continuously operating MFC system.
